# Developing a Community of Scholars for Students, Residents, and Early-Career Researchers: MedEd Collaborative—A Health Professions Education Research Collaborative

**DOI:** 10.1097/ACM.0000000000006040

**Published:** 2025-04-01

**Authors:** Matthew H.V. Byrne, Megan E.L. Brown

**Affiliations:** **M.H.V. Byrne** is a DPhil candidate, Nuffield Department of Surgical Sciences, University of Oxford, Oxford, United Kingdom; ORCID: https://orcid.org/0000-0002-2414-352X.; **M.E.L. Brown** is a senior research associate in medical education, School of Medicine, Newcastle University, Newcastle, United Kingdom; ORCID: https://orcid.org/0000-0002-9334-0922.

## Abstract

**Problem:**

Students, residents, and early-career researchers (ECRs) have limited opportunities for early involvement in high-quality health professions education research (HPER). This project aimed to create a community of scholars for medical students, residents, and ECRs to increase early-career HPER collaboration. A community of scholars is a community of practice in which the common area of interest is scholarly work. This article describes how MedEd Collaborative was established as a permanent national HPER collaborative led by medical students, residents, and ECRs.

**Approach:**

MedEd Collaborative was formed in September 2020, consisting of a committee of medical students, residents, and ECRs who oversee collaborators in the United Kingdom. Guidance on creating research collaboratives, developing a community of scholars, and collaborative writing was followed. The primary measurable outcome was to publish one original research article that used a collaborative research approach and incorporated theory. The community was cultivated by providing opportunities for early-career involvement in collaborative HPER projects and mentorship and training in HPER methods.

**Outcomes:**

MedEd Collaborative has developed a community of scholars that increased opportunities for early involvement in high-quality HPER for 82 medical students, residents, and ECRs. The collaborative structure facilitates increasing legitimate peripheral participation in HPER: acting as a collaborator provides basic research skills development, and learners can gradually assume more responsibility as their skills progress by acting on project committees. MedEd Collaborative’s research outputs progressed HPER by using conceptual frameworks to explain student volunteering decisions and experiences during the COVID-19 pandemic, and the scholarly output included 15 publications (of which 4 were original research), 19 presentations, 4 prizes, and 2 grants.

**Next Steps:**

To ensure the sustainability of the collaborative, the collaborative will refine its identity in the HPER landscape, expand the model with other methods and to other professions, strengthen its collaborative structure, and establish formal partnerships.

## Problem

High-quality, robust health professions education research (HPER) plays an important role in advancing educational practices and improving patient care. However, ineffective educational practices often persist. Reasons for this include limited HPER and good practice dissemination in some areas, including collaborative initiatives.^1^

Addressing these challenges requires a concerted effort to engage medical students, residents, and early-career researchers (ECRs) in HPER from the outset of their careers, so they can act as drivers for continuous improvement.^2,3^ However, there are often limited opportunities for learners to become involved in high-quality HPER and consequently to develop the research skills that will support them to become HPER leaders and advocates.^2,4^ Known barriers include limited funding, support and mentorship, and time within training to develop these skills.^4^

In clinical fields, research collaboratives led by medical students, residents, and ECRs have demonstrated the ability to conceive, coordinate, and publish impactful research, reduce barriers to earlier involvement in higher-quality research, and provide opportunities to develop research skills and networks.^5^ Importantly, the learning opportunities provided by research collaboratives are different from taught research modules as individuals actively participate in scholarly work, including research design, implementation, evaluation, and publication, rather than simply studying for an exam or assignment.^5^

Inspired by this innovation within clinical research and the need to increase early-career HPER collaboration, we sought to create a community of scholars for medical students, residents, and ECRs interested in HPER. A community of scholars is a specific type of community of practice, a concept developed by Lave and Wenger^6^ in which participants engage in collective learning in a shared domain. Within a community of scholars, the common area of interest driving learning is scholarly work.^7^ The benefits of being involved in a community of scholars include a supportive mentoring environment for inexperienced but interested members, mixing of diverse perspectives and interests, and opportunities to develop skills, networks, and outputs.^7,8^ Similar initiatives in HPER are often transient, are from a single institute, focus on education rather than HPER, or are faculty led.^2–4,9^ In this article, we describe how MedEd Collaborative was established as a permanent national HPER collaborative that is led by medical students, residents, and ECRs.

## Approach

### Conceptualization

MedEd Collaborative was inspired by both a need to increase research capacity at a local level for research projects into medical student volunteering during the COVID-19 pandemic and prior personal experience of the educational benefits of being a collaborator (M.H.V.B., J.A.) and leading (M.H.V.B.) clinical research collaboratives (for timeline, see Supplemental Digital Appendix 1 at http://links.lww.com/ACADMED/B693). MedEd Collaborative was founded by M.H.V.B. in September 2020, following guidance on creating research collaboratives by Dowswell et al^5^ and later guidance by Ramani et al on developing a community of scholars^8^ and collaborative writing.^7^ We outline how this guidance was incorporated into MedEd Collaborative’s approach in Table 1 and in the sections below.

**Table 1:** Goals and Steps of the Collaborative Research Approach Used by MedEd Collaborative to Oversee Collaborators in the United Kingdom, September 2020^a^

Goal	Steps
**The team**	
Clear leadership	• When the collaborative was founded, the steering committee developed a vision document as a team. This document outlined the aims and broad steps that the collaborative needs to take to progress; the document is reviewed annually. • The steering committee is overseen by a chair (M.H.V.B.), and leadership responsibilities for each output were agreed on in advance and shared among different authors. • Decisions are made by consensus among members of the steering committee who are affected by those decisions. Any disagreements are resolved by the chair.
Efficient management	• The steering committee agrees as a group how work is divided, and subcommittees are formed, accounting for individual strengths, interests, and existing commitments. • At the start of each project a protocol is written and a project timeline is developed. • Monthly meetings are held using online video conferencing software to check in on progress and to rebalance work as necessary.
Psychological safety	• Feedback on processes is collected regularly, and the group discussed members’ other commitments so that members did not feel overburdened by deadlines alongside other clinical and academic commitments. This was particularly important during the COVID-19 pandemic.
Diversity prioritized within the team	• Students, residents, and early-career researchers were selected from a range of backgrounds and specialties and stages and selected for a broad range of skills. The steering committee reflected on how individual’s skills and experiences might strengthen the collaborative. • MedEd Collaborative tried to recruit collaborators from all U.K. medical schools and had representation across 37 of 42 U.K. medical schools. This approach allowed the collaborative to recruit participants more effectively from a wider range of medical schools and to allow dissemination of research project results to students more effectively through local communication.
**Individual members**	
Motivate members by choosing a relevant and important topic	• The COVID-19 pandemic started shortly before the collaborative was founded. The collaborative included final-year medical students who provided voluntary clinical assistance and those involved in teaching medical students. As such, the projects developed explored medical students’ experiences of providing assistance because this was a relevant and important topic to the collaborative’s members.
Give members autonomy over parts of the project	• Each individual’s strengths and interests were discussed to determine how work was divided. Each member of the team was responsible for their own part of the project. For example, one person led the data collection, another the qualitative analysis, and another the quantitative analysis.
Embrace a growth mindset	• Experienced members within the steering committee offered support and mentorship for less experienced members, and subcommittees were structured so that less experienced members were grouped with more experienced individuals. • MedEd Collaborative produced a series of educational seminars on the methods used within its projects to educate collaborators about the approaches that were being used to help them move toward the center of community of practice. These seminars were delivered by members of the steering committee who had formal training in these research methods.
**Scholarly output**	
Ensure work is globally relevant and addresses an important topic	• When MedEd Collaborative started its research projects there was limited information about medical student clinical assistance during the COVID-19 pandemic, and it was an important global topic.
Deliver the output	• The primary output of the collaborative was scholarly output. This output enabled meaningful participation within the project, familiarized collaborators with the processes involved in producing a publication from start to finish, and enabled identity formation as an academic clinician.
Agree authorship order at the start of the project	• The steering committee discussed the types of collaborative authorship models with existing clinical research collaboratives. The committee tried various options during the past 3 years. • Ultimately, the steering committee opted for mainline authors for those on the steering or writing committee followed by a group author, MedEd Collaborative, for individuals who contributed from the collaborative network.
Multiple rounds of peer review	• The outputs of MedEd Collaborative projects were produced by a writing team followed by review by the wider steering committee, advisory board, and collaborators. • Meaningful feedback improved the quality of the scholarly output and developed the research skills of those involved.
**Challenges in building the collaborative**	
Ethics	• Navigating the ethical processes for research projects with authors from multiple institutions can be complex and time-consuming. • To address this, the collaborative factored in additional time required in its project development timeline.
Changing institutions	• Some collaborators moved jobs to different institutions, which created challenges in maintaining communication, continuity, and coherence in projects. • To mitigate this, the collaborative collected noninstitutional email addresses as well as institutional email addresses.
Exams and training	• Balancing research activities with clinical jobs, exams, and personal life demands, while keeping projects moving to deadline was challenging. • The collaborative helped reduce the impact of this through regular communication with team members and redistribution of work.
Distance working	• Although technology is useful at facilitating a virtual community of practice, it is not as effective for cultivating social interactions. • Members met at an annual U.K. medical education conference to increase social interactions.
Starting skill disparities	• Variations in research skills and experience levels among members required additional training and mentorship efforts, which took time.

^a^In this table, the authors describe how their approach aligns with the collaborative writing approach described by Ramani et al,^7^ which divides successful collaborative writing by 3 lenses: the team, individual members, and scholarly output.

The approach used to develop a community of scholars is conceptually rooted in Lave and Wenger’s communities of practice theory,^6^ which emphasizes the importance of social connection and shared practice in driving learning within a community. Central to this theory is the concept of legitimate peripheral participation in which newcomers become part of the community and gradually move toward full participation as they gain experience and knowledge.^6^

### Formalizing MedEd Collaborative’s structure

**Figure 1:**
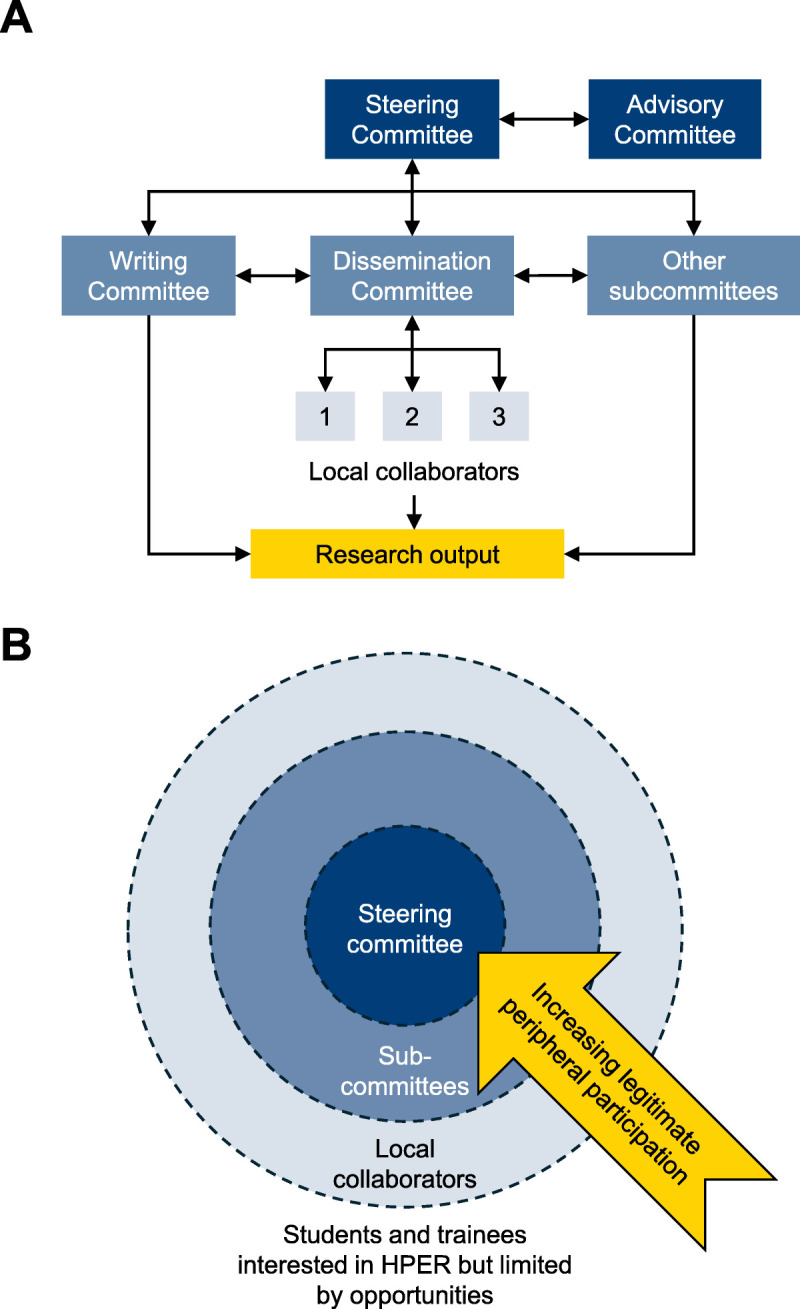
Structure of MedEd collaborative: a research collaborative used to oversee collaborators in the United Kingdom, September 2020. Abbreviation: HPER, health professions education research. (Panel A) The collaborative is led by a steering committee of medical students, residents, and early-career researchers, who oversee subcommittees and a network of collaborators and who are supported by a senior advisory committee of faculty members who provide feedback. (Panel B) The structure of MedEd collaborative facilitates legitimate peripheral participation within a community of scholars. Legitimate peripheral participation is a concept proposed by Lave and Wenger^6^ that describes the process whereby newcomers to a community engage in simpler, less central tasks within a community. Over time, as they gain experience and knowledge, they move toward more complex and central roles. The collaborative facilitates legitimate peripheral participation by providing opportunities to involve these individuals in foundational research activities (e.g., acting as a collaborator) and supports them to gradually assume more responsibility as their skills and interest develop (e.g., acting on subcommittees).

A student-, resident-, and ECR-led collaborative structure^5^ was chosen to maximize the learning opportunities available for individuals with different levels of experience.^7,8^ The collaborative is led by a steering committee of medical students, residents, and ECRs from the United Kingdom (list included at the end of this article), who conceive and coordinate projects and all parts of scholarly outputs. Within the steering committee, there are subcommittees responsible for research tasks (e.g., coordinating projects, data analysis, and academic writing). The steering committee coordinates a network of student and resident collaborators at universities in the United Kingdom, who are primarily responsible for data collection (Figure 1A). The steering committee is supported by an advisory committee of senior medical education academics (list included at the end of this article). This group provides feedback on prioritizing ideas, methods, and outputs, as well as access to a wider network and funding opportunities.^5^

### Recruitment of members

Diversity is important for collaborative projects^7^; therefore, steering committee members were recruited to represent a range of HPER interests and skills, backgrounds, stages, specialties, and institutions. We have provided a detailed reflexivity statement and demographics information in Supplemental Digital Appendices 2 to 7 (at http://links.lww.com/ACADMED/B693).

To build our community,^5,8^ we recruited collaborators for each project via social media and emails to medical schools. Collaborators from previous projects were invited to future projects, and new steering committee members were recruited through a written application and interview, which has allowed the most engaged collaborators to increase their participation^6,7^ within MedEd Collaborative.

### Deciding MedEd Collaborative’s shared purpose

To develop a clear vision for MedEd Collaborative,^8^ the steering committee discussed and agreed on its aims at the start of the collaborative, which are to increase early exposure to HPER, provide mentorship and training in HPER methods, and improve the quality of research outputs from students, residents, and ECRs.

### Ensuring quality of ideas and outputs

Project ideas were based on lived experiences within our diverse steering committee (see Supplementary Digital Appendix 2 at http://links.lww.com/ACADMED/B693); the need for the projects and their priority were determined by reviewing existing literature^7^ and feedback from the advisory committee. The collaborative’s work was informed by conceptual frameworks, and MedEd Collaborative’s research protocol was externally reviewed by the UK Medical Schools Council Education Leads Advisory Group as well as a university research ethics committee. MedEd Collaborative sought and received national endorsement for projects^5^ from the Royal College of Surgeons of England, the Royal Society of Medicine student members, and InciSion UK. These steps helped the collaborative answer research questions that were meaningful and applicable to a wider audience, and used appropriate methods.^7^

### Mentorship, training, and collaborative authorship

To cultivate our community,^8^ individuals participated in collaborative research projects.^6,7^ Specific roles within subcommittees were agreed on at the start of each project, considering individual strengths, interests, and existing commitments, and members could be part of multiple subcommittees within the same or concurrent projects. Subcommittees were structured so that less experienced members were grouped with more experienced individuals to provide mentorship.^7^ In addition to this, steering committee members with formal HPER training delivered educational seminars on the methods used for each project to educate collaborators to help them move toward the center of our community of practice.^6^

A collaborative authorship model was used because the shared benefit that individuals derive from collaborative projects motivates individuals toward the success of the project and recognizes their contributions through collaborative authorship.^5^ The contributions of collaborators are recognized under the group authorship MedEd Collaborative, with all collaborators being PubMed citable in line with the National Research Collaborative authorship guidelines.^10^ To reflect the additional work required by committee members, these authors are recognized as primary authors.^7^

### Using technology to facilitate collaboration

Because MedEd Collaborative spans across an entire country, free technology was used to efficiently administer projects.^5^ WhatsApp and email were used for written communication, Google Hangouts for video communication, Google Docs to take minutes, Google drive for collaborative cloud storage, and Zotero as a shared reference manager that integrates with Google Docs for collaborative writing. A website (www.mededcollaborative.org) was created through Google Sites.

### Outcomes

To assess the collaborative, we used the publication of one original research article in a reputable HPER journal that used a collaborative research approach and incorporated theory as the measurable primary outcome. Our secondary measurable outcomes were to publish secondary research (e.g., a review), commentary as a group on recently published research, and presentations at conferences.

## Outcomes

MedEd Collaborative has created a community of scholars for medical students, residents, and ECRs to develop skills in HPER and to allow earlier participation in high-quality research. In 3 years, MedEd Collaborative has written 15 publications, including 4 original research articles, 8 letters, 1 systematic review, and 1 protocol; the collaborative has presented 13 oral presentations and 6 poster presentations and has been awarded 4 international prizes and 2 research grants. A detailed list of the collaborative’s scholarly output can be seen in Supplemental Digital Appendix 8 (at http://links.lww.com/ACADMED/B693). MedEd Collaborative’s projects explored experiences of medical students providing voluntary clinical assistance during the COVID-19 pandemic and involved more than 2,600 participants. The collaborative’s research progressed HPER by using conceptual frameworks to explain student volunteering decisions and experiences and produced recommendations for education and health care practitioners. Each project lasted approximately 1 to 2 years from conception to preprint publication.

A total of 82 medical students, residents, and ECRs have been involved in our projects, including 24 on steering or subcommittees, and a network of 58 collaborators across 37 of 42 U.K. medical schools. Members of the collaborative have gained experience of the research process from conception to final publication, including literature review, focus groups, survey development and dissemination, obtaining ethical and local approvals, analysis of qualitative and quantitative data, and manuscript submission and revisions. Across all publications, 85.6% of mainline authors have been students, residents, or ECRs (median, 100%; interquartile range, 81.8%–100%; see Supplemental Digital Appendix 7 at http://links.lww.com/ACADMED/B693). Across the 24 members of steering or subcommittees, 17 have subsequently obtained formal research positions (other preliminary longitudinal data can be seen in Supplemental Digital Appendixes 9 and 10 at http://links.lww.com/ACADMED/B693).

MedEd Collaborative is a route for individuals who are interested in participating in high-quality HPER but lack opportunities. In Figure 1B, we illustrate how the organizational structure of the collaboration provides routes to increasing participation by legitimate peripheral participation. The collaborative provides an opportunity to involve individuals in foundational research activities (e.g., acting as a collaborator) and supports them to gradually assume more responsibility as their skills and interest develop (e.g., acting on subcommittees). In doing so, MedEd Collaborative has developed a community of scholars that widened access to high-quality HPER opportunities for students, residents, and ECRs; aided development of those individuals’ research competencies; built research capacity; and worked together to produce high-impact outputs.

## Next Steps

Through the collaborative’s scholarly work, we have successfully established our student, resident, and ECR research collaborative in HPER as a community of scholars. We now want to ensure our research collaborative can sustainably continue and grow.^8^

### Refine our identity in the HPER landscape

We want to determine how a student, resident, and ECR research collaborative can best contribute to HPER. We will determine this through an engagement process with stakeholders. Our measured outcomes of success have been focused on scholarly output. However, we also want to evaluate the impact of the collaborative on the individuals involved, considering the longitudinal impact on their careers incorporating Lave and Wenger’s theory of legitimate peripheral participation. These 2 factors will allow us to develop our framework for identifying the most impactful research questions the collaborative can answer in a way that reflects the best approach for providing opportunities for students, residents, and ECR to progress as researchers.^8^

### Expand the model with other methods and to other professions

COVID-19 provided clear research questions to answer but limited the scope of methods we could use. We plan to develop strategies to apply the student, resident, and ECR research collaborative model to educational methods beyond survey-based research and secondary research and to expand the model to include collaborators from health care professions other than medicine. To achieve this, we have started an interview-based project exploring the differences in experiences of service and learning between medical students and nursing students, and we are developing a second project based on document analysis.

### Strengthen our collaborative structure

It is time-intensive recruiting new collaborators for each project, and we want to recruit engaged collaborators who are already well integrated within their student body to increase reach. As such, we plan on approaching existing societies at each university to develop partnerships and recruit members from their societies for future projects. We currently use a document that sets out our vision for the collaborative, which was agreed on as a team to guide our actions. We plan on formalizing this into a governing document (i.e., a constitution).

### Establish formal partnerships

We want to establish formal partnerships with nationally established groups. In addition to generating new research opportunities, it could increase the resources available, including grant opportunities, expertise, and research infrastructure,^7^ and educational modules on research methods, which we could provide to our collaborators. To this end, we have already joined the National Research Collaborative—a network of student and resident research collaboratives in the United Kingdom that provide support to each other. We also liaise with senior members of the National Institute for Health and Care Research Incubator for Clinical Education Research for advice.
